# Lingual Osseous Choristoma of the Tongue Base: Unusual Presentation of a Rare Entity

**DOI:** 10.1155/2017/3234086

**Published:** 2017-10-22

**Authors:** Matthew J. Heinz, Scott M. Peters, Salvatore M. Caruana, Angela J. Yoon

**Affiliations:** ^1^Columbia University College of Dental Medicine, New York, USA; ^2^Division of Oral and Maxillofacial Pathology, Columbia University Medical Center, New York, USA; ^3^Department of Otolaryngology, Head and Neck Surgery, Columbia University Medical Center, New York, USA

## Abstract

Osseous lesions of the tongue, also referred to as osseous choristomas, are benign growths of bony tissue. These lesions are not true neoplasms but rather represent growth of normal tissue at an abnormal location. Clinically, they appear as exophytic masses of the tongue, and they are treated by surgical excision. Lingual osseous choristomas are rare entities, with only 71 reported cases in the literature. We present the case of a lingual osseous choristoma of the tongue base in a 21-year-old female. Of the cases of lingual osseous choristoma reported in the literature, ours is only the fifth case to involve this location.

## 1. Introduction

Osseous lesions of the tongue are an extremely rare benign entity, of which there have only been 71 documented cases [1–4]. Initially referred to as lingual osteomas, these lesions have since been reclassified as osseous choristomas [1]. This change in terminology reflects the current opinion that these are not true neoplasms but rather growths of normal tissue at an abnormal location. Lingual osseous choristomas appear clinically as exophytic masses of the tongue, and they are treated by surgical excision [1]. Herein, we present the case of a 21-year-old female with an osseous choristoma of her tongue base. Of the cases of lingual osseous choristoma reported in the literature, the tongue base has only been involved in four additional cases [1, 3–6].

## 2. Case Presentation

A 21-year-old female was referred to the ENT department at Columbia University Medical Center for a “bothersome” lesion of her tongue. The patient's medical history was noncontributory; however, she did report that she was a current everyday smoker. No history of intraoral trauma was reported.

**Figure 1:**
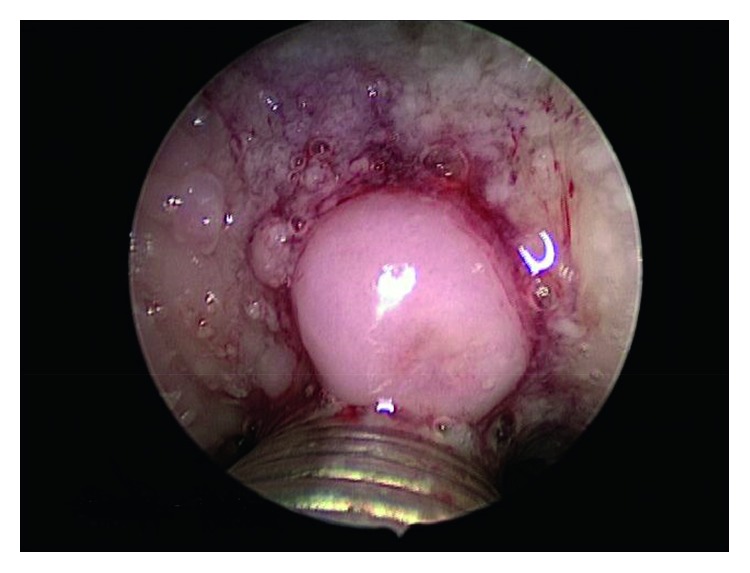
Smooth, dome-shaped mass of the tongue base identified on fiberoptic examination. The mass measured approximately 4–5 mm in size.

**Figure 2:**
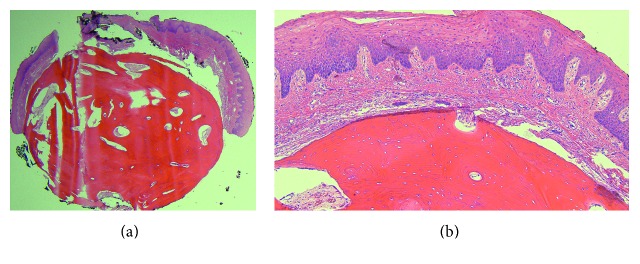
Lingual osseous choristoma. (a) Low-power photomicrograph demonstrating a nodule of dense cortical lamellar bone underlying benign-appearing stratified squamous epithelium, H&E ×20. (b) Despite its improper location within the subepithelial tissue of the tongue, the bone appears histologically unremarkable with a normal distribution of osteocytic lacunae and haversian canals, H&E ×100.

Fiberoptic examination revealed a pedunculated, smooth, dome-shaped mass of the tongue base. The mass measured approximately 4 to 5 mm in size and was located right of the midline (Figure 1()). The remainder of the tongue appeared unremarkable. Surgical excision of the lesion via carbon dioxide laser under general anesthesia was performed both for alleviation of the patient's symptoms and for histologic diagnosis. Histologic examination revealed a nodule of dense cortical lamellar bone with minimal fibrofatty marrow underlying benign-appearing stratified squamous epithelium (Figure 2()). These findings were consistent with a diagnosis of lingual osseous choristoma.

At the time of writing, the patient is one month post surgical treatment. At the one-month clinical follow-up visit post surgery, the patient was symptom free and seemed to be recovering well. She will be reevaluated again at 3 months post surgery.

## 3. Discussion

Including the current case, there have only been 72 reported instances of lingual osseous choristoma [1–4]. Gorini et al. provide a comprehensive review of 67 of the previously documented cases [1]. The authors found a wide age range among the 67 cases (five to seventy-three years of age), but the majority presented during the second or third decade of life. A strong female predilection was also reported (M : F; 16 : 44). The most frequent affected site was the posterior dorsal tongue near the circumvallate papillae [1]. Since their review in 2014, only four additional cases of lingual osseous choristoma were reported, excluding the current case [2–4]. Adhikari et al. described two cases occurring on the dorsal tongues of a 15-year-old female and a 21-year-old female [2]. Davidson et al. reported a case occurring on the tongue base in an 11-year-old male [3]. Ginat and Portugal also documented tongue base involvement in a case involving a 33-year-old female [4]. Our case represents only the fifth report of lingual osseous choristoma presenting in this particular location (two of the 67 cases reviewed by Gorini et al. also occurred on the base of the tongue) [1]. A complete listing of previously documented base of tongue osseous choristomas can be found in Table 1.

Lingual osseous choristomas vary in size from 3 mm to 5 cm and present clinically as hard masses that can be either pedunculated or sessile. In most cases, the covering mucosa exhibits a normal clinical appearance [1, 7]. Lesions may be asymptomatic, but oftentimes, patients complain of choking, gagging, nausea, or dysphagia. Histologically, a well-circumscribed, lamellated mass of vital bone is seen underlying benign-appearing stratified squamous epithelium [7, 8].

The etiopathology of this entity is still unknown, but several theories have tried to explain its pathogenesis. Two hypotheses in particular are widely recognized. The malformation hypothesis argues that the lesion arises as undifferentiated mesenchymal cells at the line of fusion between the developing first and third branchial arches [1, 8]. On the other hand, the traumatic hypothesis proposes that constant irritation of the tongue can lead to an osseous lesion occurring as a reactive or posttraumatic center of ossification [8].

The differential diagnosis of osseous choristoma is large and may include benign tumors of nerve or soft tissue, thyroglossal duct cyst, lingual thyroid, mucocele, pyogenic granuloma, and malignant tumors [7, 9]. However, one must consider the particular site of involvement when developing a differential diagnosis, as some of these lesions are more or less likely to present in certain areas. Surgical excision by scalpel, laser, or electronic scalpel is the most common treatment for osseous choristomas. Recurrence or malignant change has not been reported [10].

## Figures and Tables

**Table 1:** Documented cases of base of tongue osseous choristoma.

Author	Age (y)/sex	Size	Symptom
(1) Davidson et al. [3]	11/M	Not reported	“Foreign body sensation”
(2) Ginat and Portugal [4]	33/F	Not reported	None
(3) Kaye [5]	26/F	1 × 1 cm	“Lump”
(4) Cabbabe et al. [6]	5/F	0.6 × 0.5 × 0.3 cm	“Lump”
(5) Present case	21/F	0.5 cm	“Bothersome feeling”

y = years old, M = male, F = female.
